# Two-stage visual speech recognition for intensive care patients

**DOI:** 10.1038/s41598-022-26155-5

**Published:** 2023-01-17

**Authors:** Hendrik Laux, Ahmed Hallawa, Julio Cesar Sevarolli Assis, Anke Schmeink, Lukas Martin, Arne Peine

**Affiliations:** 1 Department of Intensive Care and Intermediate Care, University Hospital RWTH Aachen, Pauwelsstreet 30, 52072 Aachen, Germany; 2 Research Area Information Theory and Systematic Design of Communication Systems, RWTH Aachen University, Kopernikusstreet 16, 52074 Aachen, Germany

**Keywords:** Health care, Biomedical engineering

## Abstract

In this work, we propose a framework to enhance the communication abilities of speech-impaired patients in an intensive care setting via reading lips. Medical procedure, such as a tracheotomy, causes the patient to lose the ability to utter speech with little to no impact on the habitual lip movement. Consequently, we developed a framework to predict the silently spoken text by performing visual speech recognition, i.e., lip-reading. In a two-stage architecture, frames of the patient’s face are used to infer audio features as an intermediate prediction target, which are then used to predict the uttered text. To the best of our knowledge, this is the first approach to bring visual speech recognition into an intensive care setting. For this purpose, we recorded an audio-visual dataset in the University Hospital of Aachen’s intensive care unit (ICU) with a language corpus hand-picked by experienced clinicians to be representative of their day-to-day routine. With a word error rate of 6.3%, the trained system reaches a sufficient overall performance to significantly increase the quality of communication between patient and clinician or relatives.

## Introduction

Intensive care units (ICUs) cater to patients whose lives are in immediate danger and require constant supervision and care from the medical staff. The length of a patient’s stay in the ICU depends on the severity of the patient’s condition and can vary from several hours to several weeks or months in extreme cases.

Respiratory failure is a common ailment in ICU patients whose spontaneous breathing is insufficient or completely failed due to various reasons. To avoid a decrease in the blood oxygen saturation of patients suffering from acute respiratory failure (ARF), they undergo the treatment of Mechanical Ventilation (MV). It works by either positive or negative pressure ventilation, where air is pushed or sucked into the patient’s lungs. In non-invasive MV, this happens through facial or nasal masks through the patient’s mouth. However, in severe cases, physicians resort to invasive ventilation methods. Two common forms of invasive MV are the insertion of an endotracheal tube through the patient’s mouth and the tracheotomy, a surgery in which a tube is inserted through a tracheal incision. Invasive MV methods result in the patient losing his voice and, in effect, the ability to use the most effective form of communication with clinicians or relatives. The difficulty in communication is the most commonly reported distressing symptom for ICU patients undergoing MV^1^ although 53.9% of MV patients were found to meet”basic communication criteria”with the patient being awake, alert, and responsive to verbal communication from clinicians^2^. Solutions to this problem are grouped under the term”augmentative and alternative communication (AAC) strategies” and divided into low-technology AAC, such as point charts or handwriting with pen and paper, and high-technology AAC facilitating communication via handheld apps, text-to-speech software or eye-tracking commands^3^. For a patient who might already be in a state of shock with the ailments under treatment, not being able to communicate effectively can worsen symptoms like anxiety, panic and distress. Ultimately, this leads to frustration for both patients and clinicians^1^ as the patient is not able to report his symptoms and needs in an unconstrained way. This situation comes with an increased chance of the patient developing the state of delirium, having severe short-term and long-term effects on the patient’s physical and mental health^4^, which adversely affects the MV dependence duration and thus causes an overall increase in healthcare costs^5^.

A French cross-sectional study reported that between 2006 and 2015, the number of patients admitted to intensive care units for respiratory infection increased 2.7-fold^6^. Estimates put the number of mechanically ventilated patients worldwide at 13–20 million per year^7^. The current Sars-CoV2 pandemic highlights the importance of mechanical ventilation in the critical care environment as it causes severe respiratory restrictions, often leading to MV treatment. Depending on the region, healthcare system and age of the patients, different studies report the share of Covid-19 patients that receive MV to 10%–60%^8–10^.

With a particular emphasis on the above-mentioned problem of MV and delirium, both in terms of the patient’s health and the economic impact, new technologies to aid patients with temporary speech impairment in communication are needed, especially since existing AAC methods are rarely used by caregivers due to time constraints, lack of tool availability, or insufficient training^3^. Given a patient that merely has problems with producing sounds but can still make lip movements, a machine-based lip-reading system could help such patients to communicate more efficiently with the medical staff, thus constituting a fast and intuitive, high-technology AAC solution that comes with small time overhead and requires little to no training for caregivers and patients to use.

From a technology perspective, the process of predicting the transcript of the patient’s speech using only the silent videos of the patient’s mouth region movements is known as Visual Speech Recognition (VSR) or lip-reading. The contribution of this work is two-fold. First, we introduce a two-stage framework for lip-reading utilizing the ground-truth audio features of the video data set as an intermediate prediction target at training time. We further introduce a custom lip-reading data set (VSRICU) recorded in an ICU setting in the University Hospital of Aachen (UKA). Studies analyzing the typical communication between patients and nurses indicate brief and informative statements about physical care, yes/no questions, reassurances and commands to be important^1^. Thus the sentence corpus is hand-picked by experienced intensive care clinicians and designed to reflect the context of essential ICU communication. Training the two-stage model on our VSRICU data set yields a trained model being able to perform free-text lip-reading to aid speech-impaired patients in a clinical setting. To compare to the baselines of existing work in this field, we further train our model to perform VSR on the GRID audio-visual corpus^11^.

The experimental evaluation conducted in this work aims to test the following statement: the proposed two-stage model is able to perform the task of lip-reading on both the GRID corpus and our VSRICU dataset with acceptable performance w.r.t. patient communication ($WER < 10\%$). In this context, we show that using low-dimensional Mel Frequency Cepstral Coefficients (MFCC) as an intermediate prediction target outperforms other, high-dimensional audio feature representations given a fixed model capacity. Further, we prove that models trained on a small dataset (such as the VSRICU dataset) can generalize well to unseen videos by using different frame augmentation techniques in combination with a CNN-based frame encoder. We further compare the performance of our two-stage architecture against an end-to-end model from literature.

## Methods

**Figure 1:**
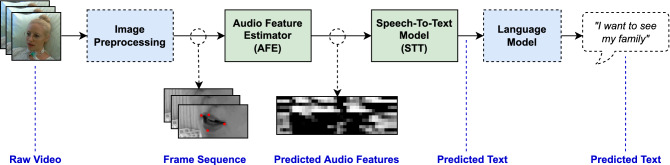
Full overview over the proposed system for Visual Speech Recognition. Single frames extracted from the source video pass through an image preprocessing pipeline followed by an Audio Feature Estimator (AFE) to infer the spoken audio features from the sequence of silent frames. The predictions serve as an input to a Speech-To-Text (STT) model for automatic speech recognition (ASR). Finally, the predicted, decoded sentences are corrected by a language model.

### Pipeline overview

In the first pipeline step, the input video is split into a series of consecutive frames, passing various preprocessing steps afterward. All images are normalized after being converted to a grayscale representation, thus removing color information. Frames are cropped to the mouth region using a face bounding box detector followed by a facial landmark detection model from the *dlib* library^12^. By using four key landmarks on the subject’s lips, all video frames are cropped to center the subject’s mouth in the image and subsequently resized to a size of $100 \times 50$ pixels. The preprocessed frame sequence is fed to an Audio Feature Estimator, a deep neural network consisting of a visual encoding stage followed by a series of recurrent layers to predict an audio feature representation from the silent frames. Here, the ground truth audio signal from the source videos is used as a target during training, but not at runtime. The predictions serve as an input to a Speech-To-Text (STT) model based on 1D-convolutional and recurrent layers performing automatic speech recognition (ASR) and using the Connectionist Temporal Classification (CTC) loss for training. Finally, a word-based language model corrects the predicted, decoded sentences. Figure 1 shows an overview over the full lip-reading pipeline.

### Data resources

#### VSRICU dataset

**Figure 2:**
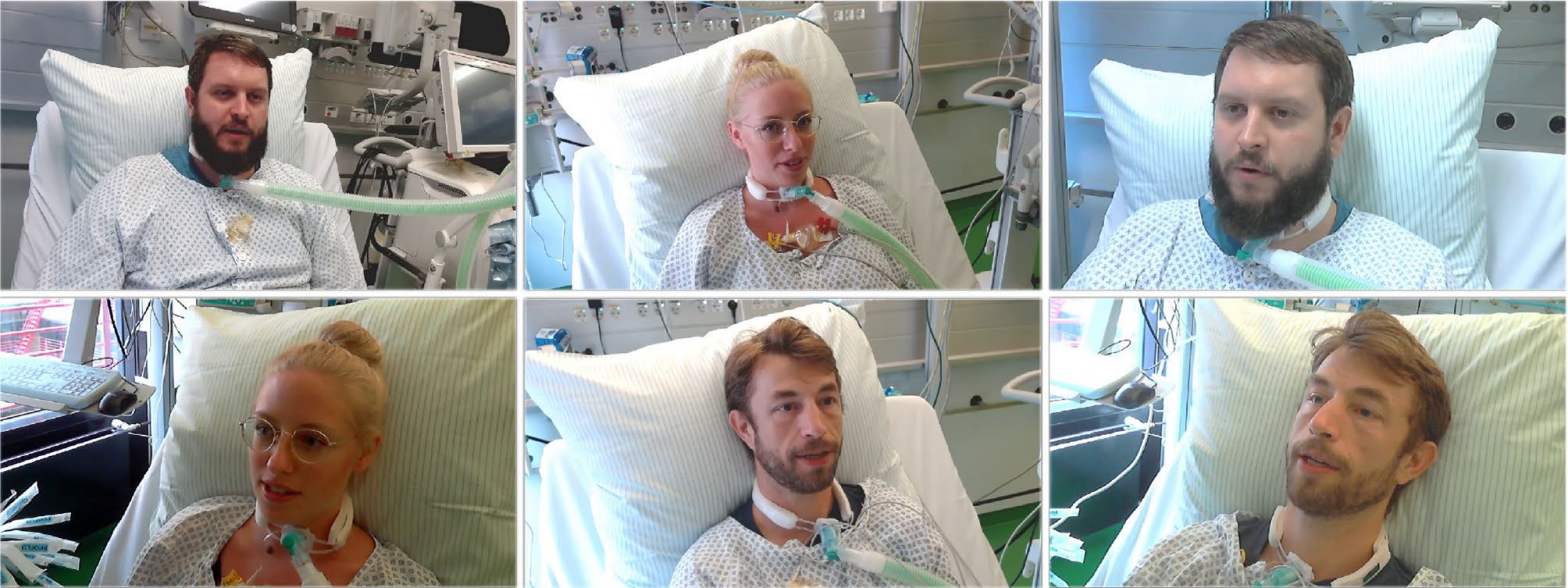
Sample frames from our VSRICU dataset, recorded at the University Hospital of Aachen.

To the best of our knowledge to date, there is no database that fits the nature of our problem statement. Various audio-visual datasets for training lip-reading systems are available, e.g. the *Lip Reading Sentences in the Wild (LRS)* datasets^13^, which have a broad and general corpus and challenging speaker movements. In contrast to the conditions found in these datasets, lip reading in an ICU setting has the advantage of a fixed camera angle, less head movement and a more narrow selection of potential conversation topics, which facilitates the training of our model with a comparably small amount of videos to ultimately perform well in the specific domain of essential patient communication. We thus recorded a new dataset dedicated explicitly to the purpose of lip reading in the intensive care setting. Our dataset, dubbed as Visual Speech Recognition for Intensive Care Unit (VSRICU), provides a total of 6900 videos of six male and female speakers (native German), each uttering 238 different sentences in German and English (around 50% German and 50% English). Recordings for the VSRICU dataset have been conducted in a room of a real intensive care unit of the University Hospital Aachen (UKA) in 2019 (pre-Covid). We used two webcams to obtain videos from two near-frontal angles being representative for a potential position of a lip reading device at the ICU bed. Frame samples from the dataset are shown in Fig. 2. The VSRICU corpus represents a selection of sentences being essential for the communication between patients and clinicians (or relatives), enabling the training of dedicated models to assist speech-impaired patients in a hospital environment. The dataset’s corpus has been hand-picked by clinicians with years of experience in the ICU environment and every sentence is recorded multiple times with each subject in the dataset.

An informed consent for publishing both the full dataset and identifying images from the dataset was obtained from all subjects and/or their legal guardian(s) in both open access and none open access publications before participating in the recording sessions of Visual Speech Recognition for Intensive Care (VSRICU) dataset. All methods were carried out in accordance with relevant guidelines and regulations.

**Table 1:** Statistics for the VSRICU and GRID datasets.

Dataset	VSRICU	GRID
# Videos	6916	34,000
# Speakers	6	34
Languages	English & German	English
Label structure	Natural, free-text sentences (1–8 words per sentence)	Artificial sentences with fixed pattern (6 words per sentence)
Video resolution	1280 $\times$ 720 px	360 $\times$ 288 px
Audio sample rate	44.1 kHz (16 kHz after resampling)	16 kHz, 44.1 kHz and 50 kHz (16 kHz after resampling)
Video frame rate	25 fps	25 fps
Video length	1–3 s	3 s
View angles	Near frontal $\times$ 2 (approx. $20^\circ$ left and right of center)	Frontal

#### GRID audio-visual corpus

The GRID Corpus^11^ contains a total of 34,000 video recordings of 34 speakers, each uttering 1000 distinct sentences. The dataset comes with transcripts of the spoken sentences to be used for training either lip reading or ASR systems. The sentences in GRID are constrained to a specified structure, with each sentence being formed as: ‘command(4) + color(4) + preposition(4) + letter (25) + digit (10) + adverb(4)’. Here the number in brackets denotes the number of options for each category (as for example [bin, lay, place, set] for the category ‘command’). Using GRID as a dataset for visual speech recognition on the one hand simplifies preprocessing by providing perfectly aligned face recordings of uniform length (3 seconds) with an excellent video and audio quality. On the other hand, the sentences spoken in GRID are not representative of real-world scenarios of a human to human communication.

Compared to GRID, the camera angle and lighting conditions in the VSRICU dataset are more challenging. A lower audio signal-to-noise ratio complicates the use of audio as an intermediate prediction target. However, the biggest challenge is given by the much smaller number of videos in the dataset (considering the training of a deep neural network model). A more detailed comparison of technical characteristics is provided in Table 1.

### Preprocessing

#### Frames

After extracting the sequence single input frames from the source video, frames are converted into grayscale reducing the images to the luminance information as shown in Fig. 1. On every individual frame, we use a face detector based on Histogram of Oriented Gradients (HOG) features combined with a linear Support Vector Machine (SVM) classifier^14^ to estimate the face’s bounding box and subsequently a facial landmark detector based on an ensemble of regression trees^15^. For both the face detector and the landmark prediction, we use the implementations provided by the *dlib* library^12^. Based on four key landmarks (left and right corner of the mouth, middle of lower and upper lip), we estimate the position of the subject’s mouth and crop the image, such that the padding (distance between mouth and border of the image) equals half the mouth width or height. Finally, the cropped images are resized to a shape of $(100,50,1) =$ (*width, height, channels*). We relinquish every video in which detectors fail to recognize the face. Before being fed to the AFE, frames are normalized to the range of $[-1, 1]$ and frame sequences are padded with black frames to have a uniform length of 75 frames.

#### Audio features

In order to facilitate the prediction of audio features from a sequence of silent frames, window size and stride are adjusted such that one set of audio features matches the length of exactly one frame. For the given datasets with a frame rate of 25 frames per second and a sample rate of 16, 000 Hz, the resulting window size is 40 ms or 640 audio samples for each set of audio features. Audio extracted from the source videos is normalized to the range of $[-1, 1]$ and afterwards transferred to the frequency domain by means of a Short-Term-Fourier-Transform (STFT) with the specific stride and window size of 640 samples using a Hann window function.

The resulting spectrum is mapped to the Mel scale, which divides the frequency domain into distinct bins of pitches using overlapping triangular windows. The Mel scale is based on the human way of hearing, as we perceive each of these bins as being equally distant in pitch. After summing the logarithmic power spectrum within all bins, a discrete cosine transform is applied to decorrelate the Mel frequency bands, resulting in a so-called *cepstrum*. Finally, Mel Frequency Cepstral Coefficients (MFCC) are given by the amplitudes of this cepstrum. Usually, the first MFCC is omitted, as it merely represents the average power of the input signal. The higher-order MFCCs are renounced as well, as they represent spectral details usually not contributing to a higher performance of the model while increasing the dimensionality of the input. For the evaluation conducted in this work, we consider both Mel Spectrograms and MFCCs as potential intermediate audio feature prediction targets. We use the *librosa*^16^ library to perform all of the above-mentioned steps in the audio preprocessing pipeline. Finally, each coefficient is normalized individually by subtracting the mean and diving by the standard deviation of the coefficient across the whole dataset, causing the preprocessed audio features to have zero mean and unit standard deviation. We choose this type of normalization over the mapping to the range of $[-1, 1]$ as it is less sensitive to outliers.

### Our model

The proposed model for visual speech recognition consists of two stages, exploiting the availability of (otherwise unused) audio data in the training set to serve as an intermediate prediction target in the lip reading pipeline. A similar approach can be found in^17^, proposing a model to map the sequence of silent frames to a mel-spectrogram reconstruction for the GRID dataset. In^18^, a pretrained speech recognition model is used in a teacher-student approach to guide the training of a visual speech recognition pipeline in order to improve performance and to yield a way of training lip reading models without ground-truth annotated video data. Similarly, in^19^ a pretrained speech-recognition model is used to provide complementary and discriminant clues for improving the training of a lip reading system.

**Figure 3:**
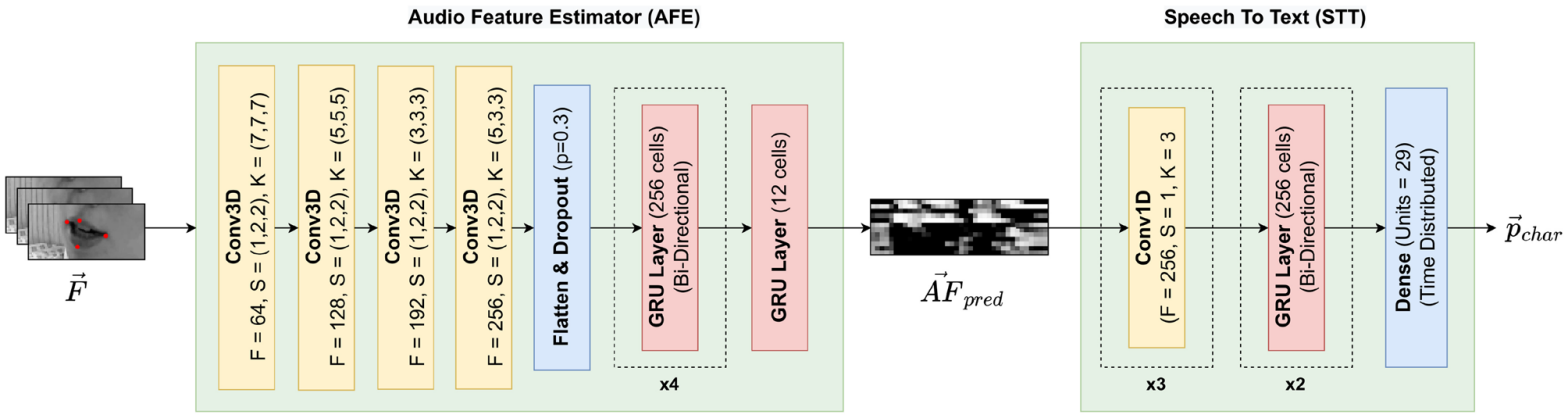
Detailed neural network architecture of AFE and STT. Important hyperparameters are provided in the layer blocks directly, where in the case of convolutional layers, *F* indicates the number of filters, *S* the stride and *K* the kernel size. All convolutional layers are followed by a ReLU-activation function omitted in the figure for readability. Identical, subsequent layers are indicated by a dashed box with the number of repetitions given below.

#### Audio feature estimator

The Audio Feature Estimator approximates the mapping from frames to audio features using a spatio-temporal feature extraction part based on Convolutional layers with a three-dimensional kernel followed by a sequence processing part with Gated Recurrent Unit (GRU) layers. In contrast to convolutional layers with two-dimensional kernels being able to learn and extract spatial features from each individual frame, the spatio-temporal (3D) convolutional layers learn a kernel performing calculations across the spatial dimensions (width and height) and the temporal dimension (across subsequent timesteps), thus allowing for learning and processing short-term temporal dependencies in the videos. The visual feature extraction part of the Audio Feature Estimator is followed by multiple Gated Recurrent Unit (GRU) layers to learn both long- and short-term dependencies between the visual encoding of the frame sequence. The output layer yields a sequence of $d_{AF}$ audio features per frame/timestep depending on the training configuration.

The Audio Feature Estimator is trained end-to-end using a pixel-wise mean-absolute-error (MAE) loss between the ground-truth and the predicted audio features. The loss function minimized for each training batch of size *N* yields

$$\begin{aligned} \mathscr {L}_{AFE} = \frac{1}{N}\sum _i^{N} \left| AF_{GT, i} - AF_{pred, i}(F_i) \right| \end{aligned}$$

where $AF_{GT}$ is the ground truth audio features calculated from each training video’s audio signal and $AF_{pred}$ is the AFE prediction given the preprocessed frames $F_i$ of the training video $i = 1, ..., N$.

#### Speech To text module

The second stage of the visual speech recognition model maps the sequence of audio features to a sequence of probabilities for each character. Input features pass through two layers of 1D-convolution to learn short-term temporal dependencies, followed by GRU layers to process short- and long-term dependencies across time. Figure 3 shows the detailed architecture for both the AFE and STT neural network models with important hyperparameters added to the layer blocks. The STT model is trained using the Connectionist Temporal Classification (CTC) loss^20^ which allows for minimizing the loss for all possible input-output alignments without the need for explicitly segmented text labels with timestamps of word boundaries. For each feature set extracted from a single video, the output layer yields a sequence of shape (75, 29) representing a probability distribution $p_t^c$ over all possible characters for each frame *t*. The set of characters (or alphabet) is composed of the 26 characters of the English alphabet along with whitespace (” ”), a padding character (<pad>) and the CTC blank token (_).

Using the CTC-loss, the STT model is trained by means of a differentiable loss function

$$\begin{aligned} \mathscr {L}_{STT} = \frac{1}{N}\sum _i^{N} -\log (p(y_{GT, i}|AF_{GT, i})) \end{aligned}$$

maximizing the log likelihood of the ground truth free-text label $y_{GT}$ given the true audio feature input $AF_{GT}$ for each sample video *i* without the need for any explicit word or character alignment information in *y*.

Besides training AFE and STT models individually and afterward evaluating them together at runtime, where the AFE output is fed to the STT model, the STT model can be fine-tuned by training on the AFE predictions instead of the ground-truth audio features of the video to obtain the modified CTC loss function

$$\begin{aligned} \mathscr {L}_{STT} = \frac{1}{N}\sum _i^{N} -\log (p(y_{GT, i}|AF_{pred, i}(F_i))) \end{aligned}$$

for optimizing the STT model parameters with $F_i$ denoting the frame sequence input to the AFE for video *i* in the batch. Though minimizing the same loss function as in the case of end-to-end lip-reading (maximizing the probability of the target sentence given a frame sequence), the substantial difference lies in the AFE already holding knowledge about the relationship between audio and video. Although the audio feature predictions are erroneous, they serve as a low-dimensional feature set encoding substantial information needed to perform a text transcription. By fine-tuning, the STT is given a chance to adapt to the errors introduced by the imperfect AFE predictions that also occur at runtime, thus improving the performance of the final, combined visual speech recognition system.

#### Language model

Sentences produced by the CTC-decoder of the STT module undergo a spell correction based on a simple bag-of-words (*1-gram*) language model, performing a word-for-word correction of the source sentence based on the total dataset’s corpus. For each word *w* in the decoded sentence, the model obtains a set of word candidates ${\mathscr {W}_d}(w)$ that originate from the source word *w* by inserting, deleting or replacing characters. At the same time, the maximum number of modifications (edit distance) is limited to $d = 2$ due to the exponential growth in candidates. The language model then selects the word with the highest likelihood of occurring in the entire corpus of the current dataset ${\mathscr {D}}$.

### Data augmentation

Considering the limited dataset size for visual speech recognition, a sophisticated visual augmentation scheme becomes essential to artificially increase the amount of available data by modifying the input data with a pipeline of random variations. This aims to increase the robustness of models against natural variations of the input data and to reduce the generalization gap between training and test performance of the model by counteracting the effects of overfitting.

The visual augmentation steps compromise horizontal flipping of images followed by an affine image transformation composed of rotation around the image center, translation, shearing and scaling. Afterward, zero-mean Gaussian noise is added to the image. Finally, the image’s luminance is randomly increased (or decreased) by adding a constant value to the image array.

All of the previous augmentation steps are applied randomly with a probability $p_{aug} = 0.8$ except for the horizontal flipping with $p_{flip} = 0.5$. Thus, not all augmentation steps are applied to all videos to further increase variation. Individual augmentation parameters are sampled from a uniform distribution for each video, but the same scheme is applied equally to all frames within a video.

### Training details

After preprocessing, input frame sequences are of shape (*N*, 75, 50, 100, 1) and the corresponding audio features are of shape $$(N, 75, d_{AF})$$ for each video of the dataset, where *N* is the batch size used for training and $$ d_{AF}$$ is the dimension of audio features considered. The sequence length for all experiments is fixed to 75, as videos from the GRID dataset are 3 s (= 75 frames @ 25fps) long by default while all videos of the VSRICU dataset are shorter and can be padded to this uniform length.

Both datasets are divided into training, validation and test set using a random split with 10% of the videos assigned to the validation and test set each. The same random seed is applied throughout the whole training procedure, meaning that all models train on the same training samples, leaving the test set unseen until the final evaluation. Both AFE and STT models are trained using an Adam optimizer ($\beta _1 = 0.9, \beta _2=0.999, \epsilon =10^{-7}$) with learning rate $1e^{-4}$ (AFE) and $5e{^-4}$ (STT) based on experimental evaluation. Except for the input data, the STT training methodology is the same for both training on ground-truth audio features and fine-tuning on predicted audio features. For the implementation of the models and the training process, we use Google’s TensorFlow library^21^.

For the audio feature representation serving as a target for the AFE training and as an input for the STT training, we compare the performance of Mel features (40, 80 or 120 frequency bins) and MFCCs (6, 12 or 24 coefficients). We further conduct all of the experiments with and without augmentation applied to the AFEs input frames. In the case of augmentation, the input frames to the AFE, when fine-tuning the STT model, are augmented as well.

To compare our model against an end-to-end model from literature, we further implement *LipNet*^22^, which is known as the first end-to-end deep neural network model for lip-reading on the GRID dataset. We see this model suited to be trained on the VSRICU dataset, as it shows good performance on the mid-sized GRID dataset while more recent end-to-end models rely on huge audio-visual resources^13,23,24^. We train *LipNet* as described in the original paper.

### Ethics

An informed consent for participating in collecting the dataset and publishing both the full dataset and identifying images from the dataset was obtained from all subjects and/or their legal guardian(s) in both open access and none open access publications before participating in the recording sessions of Visual Speech Recognition for Intensive Care (VSRICU) dataset. All methods were carried out in accordance with relevant guidelines and regulations. This is not a medical study, and it did not involve any patients, nor did it compare, assess or evaluate any kind of human behavior.Thus the need for ethics approval is deemed unnecessary according statutes of the ethics committee of the faculty of medicine of the RWTH Aachen version dated 20.03.2008, and declared in the official notice number 2020/103. The procedure of data collection, access, deletion, storing and processing has been acknowledged by University Hospital Aachen legal department.

## Results

**Table 2:** Best character (CER) and word (WER) error rates on the GRID and VSRICU dataset with and without using the spell language model after CTC decoding.

		Language model		w/o Language model	
		CER (%)	WER (%)	CER (%)	WER (%)
GRID	Fine-tuning	2.2	4.6	2.3	5.1
	w/o Fine-tuning	3.1	7.1	3.0	7.4
VSRICU	Fine-tuning	4.3	6.3	3.8	7.3
	w/o Fine-tuning	18.2	23.8	18.4	28.1

“w/o Fine-Tuning” indicates the STT model not being fine-tuned with the predicted audio features from the AFE.

The results in Table 2 show that the proposed approach to visual speech recognition is able to train a model with low error metrics for both our VSRICU and the GRID data set. All metrics are evaluated on a separate test set of unseen videos, which have not been used for AFE training, STT training or potential finetuning of the STT. For the case of the VSRICU data set with a corpus of limited context tailored to the clinical environment, the simple word-distance-based language model proves to significantly reduce the share of misclassified words in the predicted sentences. Fine-tuning the pre-trained STT on the predicted audio features clearly reduces the prediction error. The effect of this extension to the training procedure is more substantial in the case of the VSRICU dataset.

Table 2 shows the best metrics achieved for all configurations of audio features and input augmentation. Without augmentation, the minimum character error rate achieved overall audio feature configuration increases from 3.8 to 25.5% for the VSRICU dataset, indicating the importance of random input variations considering the small number of videos. We further observe that fine-tuning the STT model decreases the performance of the composed system when augmentation is not applied. A possible explanation is the STT model further overfitting the non-augmented AFE predictions in this case. In all scenarios, the performance using Mel features between AFE and STT is worse than with MFCCs. For the fine-tuned STT model on VSRICU, Mel-based systems reach an average CER of 13% while using MFCCs results in an average CER of 5.5%. While there are hardly any differences observable between the Mel configurations with 40, 80 or 120 bands, the CER tends to be lower when using more MFCCs. Consequently, combining a fine-tuned STT model with input frame augmentation and 24 MFCCs yields the best performance on the VSRICU dataset. Training the end-to-end model *LipNet*^22^ on the VSRICU dataset results in a CER/WER of 7.2%/12.7% (with language model), which is significantly worse, while the performance on GRID is reported to be similar with a CER/WER of 1.9%/4.8%.

**Table 3:** Samples of misspelled predictions of the full pipeline.

	Ground-truth text	Prediction (before LM)	Prediction (after LM)
GRID	Set red in **s** four now	Set red in **n** four now	Set red in **n** four now
	Lay blue **in** j two soon	Lay blue **an** j two soon	Lay blue **n** j two soon
	Bin red at **s** three again	Bin red at **f** three again	Bin red at **f** three again
VSRICU	I feel **happy**	I feel **hppy**	I feel **happy**
	I **am hot**	Im **hoot**	Im **hot**
	Im **hungry**	Im **hngry**	Im **hungry**

Ground-truth labels are shown in the left column, raw predictions by the STT module and the language model output are shown in the middle and right column.

From the text predictions on both datasets, it becomes obvious that short words (and sentences in the case of VSRICU) are among the more commonly misspelled. The prediction errors on the GRID dataset are limited to almost exclusively the single letters present in every GRID command. This type of error is unfeasible to correct for a word-level language model (and due to the structure of the GRID corpus for any other language model), thus explaining why the effect of using a language model here is not visible in the evaluation metrics. Further we avoided the use of a higher order language model (bi-gram, tri-gram) due to the limited corpus available for training it on the VSRICU dataset. Table 3 shows a selection of erroneous predictions made by the pipeline for both datasets.

**Figure 4:**
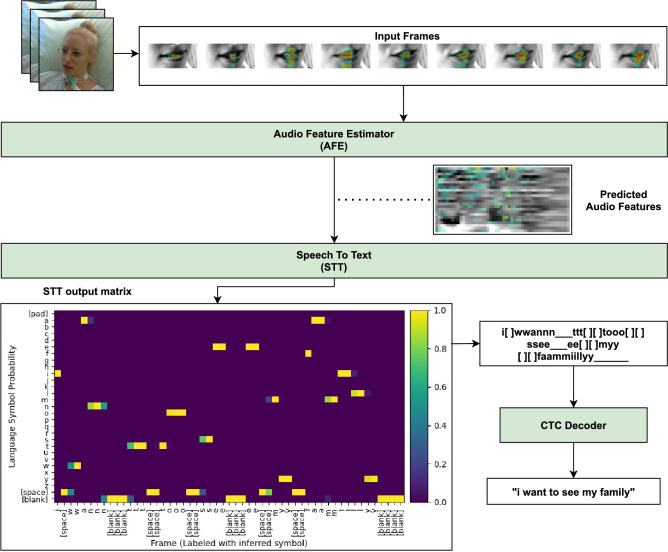
Overview over the fully trained system at runtime. Input frames are fed to the AFE to predict the video’s audio features. The STT model uses the audio feature prediction to infer a probability distribution over the set of possible language symbols for each frame, which are decoded to spoken sentences. The lip images in the top part of the figure show a subset of the video’s frames with a gradient cam^25^ overlay, indicating which parts of the image contribute to the prediction of the AFE. Similarly, the saliency map overlay on the audio feature prediction indicates which coefficients in the audio features contribute most to the text prediction of the STT.

Figure 4 visualizes the signal flow through the full pipeline composed of image preprocessing, audio feature prediction, speech-to-text conversion and CTC decoding. Similar to the technique used in^26^ or^25^, we back-propagate class decision boundaries to the network input to visualize the region of interest in the input signal. The colored regions in the input lip frames prove the model to work and perform visual speech recognition. Looking at the intermediate audio feature signal, we can see that lower-order features contribute more to the character sequence output than the higher-order features. This can be expected due to the lower-order features carrying more information after the DCT is performed to obtain MFCCs. The lower part of the figure shows the sequence of character probabilities before being processed by the CTC decoder.

## Conclusion

This work introduces a two-stage approach to Visual Speech Recognition in an intensive care environment using low-dimensional audio features as an intermediate prediction target. The fully trained system achieves a word error rate of 6.3%, which we assume to be sufficient to restore the essential patient-to-clinician communication for speech-impaired patients. The experimental evaluation in this work suggests that both careful data preparation (incl. augmentation) and postprocessing of text predictions play a vital role in the final system’s performance.

The recorded sentences in our ICU dataset are closer to the context of a patient-clinician communication than existing datasets. Nevertheless, the linguistic diversity of the corpus is still limited to the essential needs of the patient. The limited context of the dataset introduces a bias towards these sentences in models trained using the recorded videos. Although this leads to higher accuracy for sentences originating from the dataset, it might worsen the predictions if the linguistic context changes, e.g., in a conversation between the patient and his/her family. Further, though planned to be expanded, our dataset still suffers from its small sample size of only a few thousand videos. This limits the capacity of the model trained using this dataset, even using techniques like image augmentation to artificially increase the dataset’s size. Finally, the reality gap is increased by the fact that all subjects in the VSRICU dataset are young and healthy adults, which implies that patients in need of the proposed system do not have problems expressing a regular lip movement. Although younger people might suffer from respiratory failure (and other diagnostic findings blocking speech utterance), the majority of patients in need of the system will not be able to move their lips vividly like a young and healthy person due to other ailments, weaknesses, or simply, age.

The limitations mentioned above can be addressed both from the data and the model perspective. On the data side, a flexible and accurate medical lip reading solution requires the extension of our VSRICU dataset both quantitatively (total number of videos) and qualitatively (number of speakers and variety of the linguistic context). On the model side, the two-stage approach allows for using other publicly available datasets to increase the prediction quality, e.g., by pre-training the second stage of the model with an extensive, established speech recognition dataset for the application in a non-clinical conversation of the patient. As for the aforementioned problem of elderly or ill people, the trivial solution of recording a dataset featuring this specific group with the desired sample size and diversity of people is not feasible for ethical reasons. Nevertheless, this problem might be addressed in a data-driven approach as well by artificially worsening the visual speaker dynamics, which mainly involves slowing down lip movement and reducing the maximum opening of the mouth to represent the lip movement and speed of elderly, weak or ill patients. While this can be implemented by resampling the video and warping frames based on facial landmarks, applying recent advances in generative models such as neural network based style transfer^27^ might facilitate more realistic results, using only a small amount of sample videos from the target group to modify the existing dataset.

Considering future work, we are currently developing a hardware prototype that will allow us to test our theoretical results in a real-world intensive care setting. This will help us understand the limitations of our approach and identify any reality gaps between our theoretical work and the actual conditions inside the intensive care unit. Besides the above-mentioned problem of the VSRICU dataset not being entirely representative of the clinical target group, working on a hardware implementation will introduce additional challenges to overcome. One of them is the real-time capability of the system, which might be improved by using state-of-the-art solutions such as the MediaPipe^28^ framework for video processing. Considering the quality of predictions on the other end of the system, we are working on a more sophisticated and adaptive language model tailored to the use with intensive care patients. The very specific application allows for a precise prediction of the most important sentences and commands uttered by the patient as reflected in our dataset. At the same time, state-of-the-art general language models can help to improve text inference in a less restricted conversation context. All of the above-mentioned points, such as the robustness of predictions, the real-time capability of the system, and the use of adaptive, state-of-the-art language models, are subject to our current continuation of the research presented in this document and will ultimately facilitate the implementation of our theoretical work in the intensive care unit.

## Data Availability

The full VSRICU dataset is available from the corresponding author on reasonable request.
